# Cardiac care for the economically challenged: What are the options?

**DOI:** 10.4103/0974-2069.52813

**Published:** 2009

**Authors:** Sunita Maheshwari, VS Kiran

**Affiliations:** Department of Pediatric Cardiac Sciences, Narayana Hrudayalaya, Bangalore, India

The hardest part of being a pediatric cardiac medical person in India is having to tell a patient the cost of open heart surgery or device closure and watch the despair in their eyes. To produce the finances required can wipe out a family. We have all encountered families where they sold their last piece of jewelry or their only piece of land to be able to offer cardiac care for the child they love. For families such as these, there are now various government programs and charities available that can help in times of need. The aim of this article is to spread the information available so that more patients can access the resources now available in our country.

One could argue philosophically that in a country that still has malnutrition and malaria, we should not even be attempting to repair hearts in children, an exercise that can be expensive and, at times, a palliative proposition. On the other hand, we just put Chandrayan on the moon. It is our opinion that as the world's largest democracy, our families have the right to be able to offer cardiac care to their children and it is our duty to provide high-quality cardiac intervention at an affordable cost to these families and their children. Nothing stops the government and pediatricians from working on the issues of malaria and diarrhea in parallel with advances in high-end medical services. It is a matter of pride that the pediatric cardiac community in India is offering such high-quality surgery and transcatheter interventions to children with heart disease that children from all over the world are coming here – Indian cardiac centers have become an oasis in a desert of poor cardiac care in this part of the world.

Thus, as a nation, having developed expertise in treating children with heart disease, including complex ones, we need to ponder on the next issue – how do we make this care affordable and within the reach of the majority, irrespective of their financial status. How can a hospital manage to achieve high volumes without imploding or compromising on patient care. It is commonly believed that operating on children with heart disease is not “profitable” and is not sustainable. We will proceed to describe below some of the methods that can be used to provide high-quality, low-cost care to children with heart disease.

1. The concept of a low-cost package system, i.e. the patient knows at admission the total cost of hospitalization with no extra charges imposed for extra days in the hospital. This immediately gives the patient a feeling of security that he will not enter with a full wallet and exit a pauper. This does mean that certain patients, who have long intensive care unit (ICU) stays, skew the cardiac surgical budget for the hospital. However, higher volume means that in the mix of cases will be patients who are discharged in 5 days so the outliers cost is made up by the “easier” cases.
2. How does one create a low-cost package, i.e. how does one perform surgery for Rs. 80,000 when everything from the pump oxygenator to the staffs' salary costs money. A high-quality sterilization system can ensure longevity of use of operation theatre (OT)/ICU items, lower salary structure for medical personnel, a standardized system of workflow, including admission/anesthesia check/on call echo with surgeon and pediatric cardiologist can lead to rapid posting of patients and the ability to turnover the OT fast. In addition, hiring of fast and outstanding dedicated pediatric cardiac surgeons with a well-equipped and exclusive pediatric cardiac ICU with dedicated pediatric intensivists ensures a good surgical outcome. A low-mortality statistic spreads like no public relation exercise ever can. A higher volume ensures a proportion of patients will need off pump surgery or closed heart surgery and the balance sheet evens out.
3. Despite a low-cost package, there are still patients who cannot afford this. Thus, the idea of a charitable wing makes sense in a country such as ours. A procedure package is given to the patients and they pay what they can afford. What they cannot afford is raised for them by the charitable wing fund raiser from kind-hearted souls and their donations. The hospital gets its package and so remains sustainable, the patient pays what he/she can afford and the donor's money is well used.
4. However, charity is not always the best way to grow and be sustainable. Thus, other models have been conceived, one of the more successful of which has been the Yeshashwini insurance scheme – each member of a cooperative group pays a nominal amount and the corpus created is used to fund surgeries that are one-time expensive medical interventions. The Yeshashwini scheme has been adopted by the Karnataka Government and has become a highly successful model of cardiac care for the poor.
5. Insurance is one way in new India that all patients will have access to high-quality medical care without burning a hole in their pockets. It is important to advise families to take insurance cover that starts at birth (so it covers neonatal cardiac lesions) as also to cover pre-existing illnesses. It is up to us, the medical community, to audit ourselves and ensure that the insurance cover is not misused, i.e. unnecessary procedures not be carried out on patients and the bills are not inflated for these patients. If hospitals get a bad reputation and insurance stops covering cardiac care, it is the patients who suffer.
6. Medical loans – another novel concept – wherein the State Bank of India (the largest state-owned bank) will now give interest-free loans to any patient for urgent surgery. This allows the family time to breath, get the intervention done without further delay and then return home to sort out the financial situation.

Finally, it is our belief that greatest good can be done by governments with a will. Insurance schemes for the poor have been implemented in states such as Karnataka, Gujarat, Maharashtra and Andhra Pradesh and other states are following suit.

Below is a list of different schemes applicable to different governments or states. This is not an exhaustive list and similar schemes are being implemented in other states and by the Central government in various degrees of public and private partnership. Any doctor/hospital willing to help these patients can access information on various government schemes and private-public partnerships by contacting the local health care department.

## KARNATAKA SCHEMES

### Yeshasvini scheme (www.yeshasvini.org)

Considered a very impressive model in which the law of large numbers is effectively used to provide a high degree of health security to the poorest populations of the world. Yeshaswini scheme has the acclaim for providing health security to large sections of the population in a developing country, depending less on the resources but more on mobilizing capacity and organization.

The Arogya Raksha Yojana was started in Anekal taluk of Karnataka in 2005, under which the insured is entitled to free outpatient treatment at 17 network hospitals, special discounts on outpatient diagnostics, hospitalization for medical conditions and 100% coverage for over 1600 surgeries, both complex and simple.

The Suvarna Arogya Chaitanya Yojana-e is another novel idea executed by the Karnataka government. It involves free treatment of cardiac ailments to all school-going children. Unlike the Yeshaswini scheme, both surgical and non-surgical modalities are undertaken through this scheme. Each child is eligible for a maximum grant of 1.2 lakhs toward the treatment cost. Also, expenditures toward travel of patient and attendant to the referral hospital are covered, along with free outpatient investigations and consultation.

The Hridaya Spandana scheme aims to support at least one surgery a day. It works by an understanding between the government, three private charitable trusts as well as a private hospital, respectively, to help poor patients undergo heart procedures.

## TAMIL NADU SCHEMES

The Government of Tamil Nadu has constituted the Tamil Nadu State Illness Assistance Society with a revolving fund of Rs. 15 crores as an initial corpus fund. The funding pattern is in the ratio of 2:1 between the State Government and the Government of India.

“Heart Surgery for School Children” is a special scheme for children aged between 5 and 15, wherein the government will meet the entire cost. The Department of Public Health, Medical Education and District Administration jointly implement the project.

In order to facilitate heart surgery for children from poor families without any delay, the Young Children Heart Surgery Scheme is being implemented by this government in partnership with private hospitals. Under this scheme, the government provides grants-in-aid up to Rs.70,000/- to private hospitals.

## UTTAR PRADESH (UP)

Rajiv Gandhi Arogya Yojana This is UP government's pilot project at Amethi for out patient care of cardiact problems.

## ANDHRA PRADESH (AP)

In 2004, the government of AP announced financial aid for the treatment of children aged 0–12 years suffering from heart diseases by utilizing facilities in government and private hospitals.

## GUJARAT

Replications and extensions of the Yeshaswini scheme have been tried in Gujarat.

Children are also referred from Gujarat at the discretion of the state-run UN Mehta cardiac center to Bangalore for advanced and complex heart surgeries. The entire cost of the surgery is borne by the state government.

## CENTRAL AND STATE GOVERNMENTS

The Offices of the Honorable Prime Minister and Chief Minister award grants toward heart surgery from relief funds. Poor patients availing general ward may benefit up to Rs. 30,000/- from the Prime Minister's relief fund and up to Rs. 25,000/- from the Chief Minister's relief fund.

## APPENDIX 1

The table below lists as many details as we could gather on various schemes/non-government organizations and other resources.[5]

**Table:**

	Community	Organizer	Insurer	Administrator	Provider	Premium	Benefit package	Risk management
ACCORD	Tribals	ACCORD	Royal Sundaram Insurance Company	ACCORD	ACCORD hospital	Rs. 30 per person per year	Hospitalization expenses up to a maximum limit of Rs. 3000. No exclusions	Collection period, salary for providers, essential medicines and STGs
Karuna Trust	SC/ST population in T' Narsipura taluk of Mysore district	Karuna Trust	National Insurance Company	Karuna Trust	Government hospitals	Rs. 20 per person per year	Medicine cost @ Rs. 50 per inpatient day. Loss of wages @ Rs. 50 per inpatient day	Collection period, flat rate
Yeshasvini	Members of the cooperative societies	Yeshasvini Trust	Yeshasvini Trust	Family Health Plan Ltd	Private hospitals	Rs. 120 per person per year	Cover for surgeries up to a maximum of Rs. 200,000 per patient per year	Collection period, only surgical conditions, preauthorization, tariffs fixed for procedures, photo id card
RAHA	Tribals	RAHA	RAHA	RAHA	Network of “mission” clinics and hospitals	Rs. 20 per person per year	Unlimited OP cover, hospitalization cover for a maximum of Rs. 1250	Collection period, salary for providers, strict referral system, copayments
JRHIS	Farmers	JRHIS	JRHIS	JRHIS	MG medical college	Rs. 100 per family per year	OP cover by VHWs, hospital cover at medical college	Family as the enrolment unit, collection period, referral system
SEWA	Self-employed women and their dependents	SEWA	ICICI–Lombard	SEWA	Public and private hospitals	Rs. 85 per person per year	Hospitalization expenses up to Rs. 2000 per patient per year	
Student's Health Home	Students	SHH	SHH	SHH	SHH	Rs. 5 per student per year	Unlimited OP and IP at SHHrun facilities	School is the enrolment unit, providers paid fixed salaries. Definite collection period, referral system, mandatory cover of the entire population
VHS	Rural population	VHS	VHS	VHS	VHS	Rs. 100 per person per year	Hospitalization expenses up to maximum limits	Nil

**Table:** Some health insurance products in the Government sector

	Community	Organizer	Insurer	Administrator	Provider	Premium	Benefit package	Risk management
Universal Health Insurance Scheme	BPL families	?	4 public sector insurance companies	?	Any hospital	Rs. 548 for a family of five (Rs. 300 subsidized by the government)	Hospitalization cover up to a maximum limit of Rs. 30,000 per family per year. Personal accident up to Rs. 25,000. Loss of wages @ Rs. 50 per patient day	Family as the enrolment unit, waiting period
Kudumbashree (proposed)	SHG members and their dependents who belong to BPL families	Kudumbashree and Govt of Kerala	ICICI-Lombard	SHGs	Empanelled hospitals	Rs. 399 per family per year, Rs. 366 subsidized by the government	Hospitalization up to a maximum of Rs. 30,000 per family per year. No exclusions. Personal accident up to Rs. 100,000. Loss of wages @ Rs. 50 per patient day for a week	Family as the enrolment unit
AP Scheme (proposed)	BPL families	AP Government	4 public sector insurance companies	A TPA	Empanelled hospitals	Rs. 548 for a family of five (Rs. 400 subsidized by the government)	Hospitalization expenses up to Rs. 25,000 for surgical conditions and Rs. 75,000 for serious conditions. But, only for the first 3 days for medical conditions	Waiting period. Copayments after 3 days for medical conditions
Karnataka Scheme (proposed)	BPL families	Karnataka Government	4 public sector companies	Department of health staff (for collection of premium)	Any hospitals, especially public sector hospitals	Rs. 548 for a family of five. Rs. 300 subsidy from the government	Hospitalization cover up to a maximum limit of Rs. 30,000 per family per year. Personal accident up to Rs. 25,000. Loss of wages @ Rs. 50 per patient day	Waiting period, family as the enrolment unit
Assam Scheme	All Assam citizens except government servants/those with more than Rs. 2 lakh per annum income	Assam Government	ICICI-Lombard				Hospitalization expenses up to a maximum of Rs. 25,000 for select disease conditions, e.g. cancer, IHD, renal failure, stroke etc	Mandatory cover of the entire population
